# COVID-19 Pandemic and Mental Health Status of Saudi Citizens Living Abroad

**DOI:** 10.3390/ijerph18157857

**Published:** 2021-07-25

**Authors:** Marwah Ahmed Behisi, Hussain M. Altaweel, Reham F. Gassas, Mansour Aldehaiman, Abdulmajeed A. Alkhamees

**Affiliations:** 1 Clinical Child Psychologist, National Center for Mental Health Promotion, Riyadh 12211, Saudi Arabia; mbhisi@ncmh.org.sa; 2 Child and Adolescent Psychiatrist, Department of Psychiatry, Prince Sultan Military Medical City, Riyadh 11159, Saudi Arabia; Hussain.m.altaweel@gmail.com; 3 Consultant of Marriage and Family Therapy, King Fahad National Guard Hospital, Riyadh 12211, Saudi Arabia; GassasRe@ngha.med.sa; 4 Consultant of Marriage and Family Therapy, Mawadah Wa Rahmah Counseling Center and Waaie Medical Center, Riyadh 13317, Saudi Arabia; msdehaiman@gmail.com; 5 Department of Medicine, College of Medicine and Medical Sciences, Qassim University, Buraydah 52571, Saudi Arabia

**Keywords:** COVID-19, pandemic, SARS-CoV-2, mental health conditions, risk factors, anxiety, depression

## Abstract

Background: The COVID-19 pandemic is a global health crisis associated with unprecedented levels of morbidity and mortality worldwide. The COVID-19 pandemic has been suggested to contribute to a great burden on global mental health. We assumed that individuals in quarantine outside their home country would be more vulnerable to developing mental health disorders during the current pandemic and might face difficulties in accessing mental health services. Aim: To explore the degree of association between the COVID-19 pandemic and mental health status of Saudi citizens living abroad. Objectives: (1) To measure the prevalence and risk factors of mental health problems among Saudi citizens studying and living abroad during the COVID-19 pandemic; (2) to assess the correlation between the COVID-19 pandemic and mental health status of Saudi citizens living abroad; and (3) to explore the level of anxiety/depression during the COVID-19 pandemic. Methods: A cross-sectional survey was conducted from August 2020 to September 2020 using a self-administrated questionnaire composed of sociodemographic, (GAD-7) and (PHQ-9) scales. Results: A total of 64% of participants experienced psychiatric symptoms during the pandemic, and 34% and 30% met the diagnostic criteria for symptoms of depression and anxiety, respectively. The risk of psychological symptoms was more likely experienced by females, young, single, or divorced, or those who were living alone. In addition, those who lived in the UK and Ireland were more likely to develop depressive and anxiety symptoms. More than 80% appreciated the response of the Saudi government and embassy to meet the MH needs of students undergoing quarantine abroad and in Saudi Arabia. Conclusions: The COVID-19 pandemic represents an unprecedented threat to global mental health. Two-thirds of study participants who were in foreign countries during the COVID-19 pandemic reported anxiety or depressive symptoms. Living away from family and friends was significantly associated with increased loneliness and psychological distress. These and other findings highlight the need to remove barriers preventing easily accessible online mental health services, social and family support, and timely provision of resources.

## 1. Introduction

Severe acute respiratory syndrome coronavirus 2 (SARS-CoV-2) was first identified in mid-December 2019 in Wuhan City, China, and the outbreak was declared as a global public health emergency on 30 January 2020, by the World Health Organization (WHO). WHO officially named the disease COVID-19 on 11 February 2020, and a pandemic was declared on 11 March 2020 [1]. Coronaviruses are a family of single-stranded RNA viruses with high mutation rates that primarily affect the respiratory system. SARS-CoV-2 is a novel strain and has approximately 79% genetic similarity with SARS-CoV-1 [2,3]. Although these viruses predominantly attack the respiratory system, they also produce a wide spectrum of clinical presentations in almost all other organs [1,4,5,6].

Many countries have reacted responsibly to novel coronavirus 2 (NCV2) COVID-19 and announced preventative steps. These steps were taken in many countries affected by COVID-19 to control human-to-human transmission [1,4,7]. From mental health (MH) perspectives, the COVID-19 pandemic and associated preventive measures including social isolation have caused increasingly severe stress on people worldwide, leading to a MH crisis and suicide epidemic [1,8,9]. Additionally, epidemics and pandemics of fatal viral infections including COVID-19 are associated with overwhelming acute and long-term psychological stress, leading to diverse mental illnesses and medical diseases [8,10]. Many of the studies in the literature were conducted among the Chinese population as they appeared to be the first and largest nation affected by this pandemic. In a review article, the authors found that the majority of published articles (18/28 of all articles; 64.3%) and all the observational studies (4/4; 100%) were from Chinese Centers [11]. It was found that 25% of Chinese college students living in different adverse circumstances developed anxiety symptoms attributed to the COVID-19 outbreak [12]. In another study involving the general population of China, more than 50% of respondents reported adverse psychological effects during the COVID-19 pandemic [13]. In this context, Brooks and colleagues suggested that the psychological impact of quarantine can be long-lasting. Therefore, health providers should ensure that this experience needs to be as supported as possible for people testing positive for COVID-19 [14]. The researchers reported a variety of psychological symptoms including phobias, anxiety and depression, suicidal ideations, and obsession and compulsion.

To reduce the risk of developing common MH conditions during the pandemic, social and family support networks, telemedicine health services, and other resources including financial support are crucial for students stranded in foreign countries. Liem and colleagues (2020) further emphasized the need for social support and outreach programs for migrant workers stranded in any host country during the COVID-19 pandemic [15]. Psychological reactions to pandemics include maladaptive behaviors, emotional distress, and defensive responses [16]. Hence, for people experiencing psychological crises concerning public health emergencies, many countries have previously developed preventive procedures and practices that are also applicable to COVID-19 [17].

The Ministry of Health in the Kingdom of Saudi Arabia (KSA) declared MH a priority in the Vision 2030 strategic plan to ensure the KSA’s future as a nation where all people thrive, live peacefully, and stay safe [18]. Saudi citizens either live abroad for education and/or work purposes and are scattered around the globe (mainly the US and UK) with an estimated number of one hundred thousands [19]. Therefore, collecting data on the MH of Saudi nationals stranded in foreign countries during the COVID-19 pandemic is an essential strategy. Another priority was to explore the short- and long-term psychological and social impact on students and employees working in foreign countries. Many studies—including surveys—have identified the psychological impacts of a lack of family support and isolation on MH. However, studies concerning people stranded in foreign countries are limited [8,13]. We assumed that individuals in quarantine outside their home country could be more vulnerable to develop depression and/or anxiety during the current pandemic and might face difficulties in accessing mental health services. To our knowledge, MH during this pandemic has received little or no research interest to date in a Middle Eastern population, particularly among Saudi citizens.

### 1.1. Study Aims

To explore the association between the COVID-19 pandemic and mental health status of Saudi citizens living abroad.

### 1.2. Objectives

1. To measure the prevalence and risk factors of depression and anxiety among Saudi citizens living abroad during the COVID-19 pandemic.
2. To assess the correlation between the COVID-19 pandemic and the mental health status of Saudi citizens living abroad.
3. To explore the level of anxiety/depression during the COVID-19 pandemic.

## 2. Methods

### 2.1. Design and Setting

This cross-sectional study was conducted using an online self-administrative questionnaire directed to Saudi citizens living abroad before March 2020.

### 2.2. Participants and Sample Size

According to the Saudi Press Agency, a total of 124,228 Saudi citizens live abroad. A total of 79,113 are Saudi students who currently study with 45,115 accompanied family members [19]. Saudi students are mainly sponsored by the Saudi government, which involves paying their tuition and receiving a fixed monthly income for themselves and their family as well as covering them with medical insurance during their stay abroad. In April 2020, the government response was swift when it launched a program for its citizens abroad in which the embassies and consulates contacted and offered an extra monthly allowance and arranged free accommodation and flights back home to any citizen abroad. Any citizen who came back home was placed in mandatory quarantine in a hotel for at least two weeks free of charge. The sample size was calculated using the SurveyMonkey website [20]. The calculated sample size was 383 based on a population size of 124,228, with a 5% of level of significance and 95% confidence interval. Overall, a total of 765 participants from within and outside the KSA met the study eligibility criteria. This sample size is almost doubled as sampling in a cross-sectional study should be inflated to account for the expected low response rate.

### 2.3. Recruitment Plan

Participants who were living outside the country during the COVID-19 pandemic before March 2020 including citizens the government evacuated were conveniently recruited online. The population was approached using access to the Ministry of Health (MOH) data record concerning Saudi citizens who stayed in quarantine after returning to the country through the airlift evacuation process and by distributing the survey via social media platform groups for people who were abroad.

Inclusion criteria were any Saudi citizen living abroad before March 2020, adult males and females with age ≥18 years old.

### 2.4. Procedure

The purpose of the study was explained in the questionnaire, and the complete package of this survey was created and distributed online by the social media channels using Google Survey Platform (Google LLC, Mountain View, CA, USA). The participants were asked to complete a self-administrated questionnaire composed of the following components: sociodemographic variables, levels of anxiety using Generalized Anxiety Disorder (GAD-7) and depression using a patient health questionnaire (PHQ-9), access to MH services, participant’s coping abilities, and their perceptions to the Saudi government response [21,22]. At the end of the questionnaire, participants were allowed to add free text with any additional information they wanted to express. Simultaneously, responders were also permitted to ask any questions if they needed any urgent access to MH support. The participants were also asked to add their emails to send more information to provide or access MH support and services in host countries around the globe. Sociodemographic data included their gender, age, education level, country of origin, country of current residence, and the number of years that they had lived in each country.

The PHQ is a 9-item questionnaire with a possible score between 0 and 27, and a cutoff score of 10 for depressive symptoms. The Arabic version of PHQ-9 is reliable and valid in clinical practice and research [21]. The GAD is a 7-item questionnaire with possible scores between 0 and 21, and a cutoff score of 10 for anxiety symptoms. Both assessment tools were found to have good reliability and internal consistency among the study population with Cronbach’s alpha of 0.85 and 0.92 for the PHQ-9 and GAD-7, respectively. Respondents were asked to express their symptoms using a 5-point Likert scale ranging from 1 (not changed) to 5 (changed completely) and the total score ranged from 1 to 5 [23]. In total, there were two scales and one self-administrated questionnaire used in this survey. The total time taken by each participant to complete these assessment tools was about 20–30 min.

### 2.5. Ethical Considerations

The research protocol was carried out according to the ethical principles of the Declaration of Helsinki and submitted to the General Administration for Medical Research and Training, MOH, Riyadh. The Central Institutional Review Board of MOH approved the protocol on 23 July 2020, log number: 20–154 M. Participants were provided with a complete description of the purposes and methods used in the study, information protection procedures and benefits of the survey, and informed consent was obtained. Participants were given contact information for the primary researcher at the end of the study, so any questions they may ask could be addressed. Participants were informed of their rights to refuse to answer any question, withdraw at any time without explanation or negative consequences, and to have their data excluded from the analysis. To keep confidentiality and enhance participation, no identifying information was requested from the participants. No risk to participants was foreseen from participating in this study.

### 2.6. Statistical Analysis

Data were entered into a Microsoft Excel spreadsheet. The data were cleaned and prepared prior to its export into SPSS software for analysis. A codebook with variables and their labels was created. Categorical variables were expressed as frequencies and percentage and summarized in tables. Relationships among variables were presented in graphs. Significant correlations were determined based on the outcomes measured (continuous or categorical) using the *t*-test and/or Chi-square correlation test. The level of significance of any possible association between variables was set at *p* < 0.05.

## 3. Results

In total, 662 participants completed the survey with adequate data included in the analysis with a response rate of 86.5%.

### 3.1. Sociodemographic Characteristics

The majority of them were between the ages of 25 and 34 (64.0%), followed by those below the age of 25 years (21.6%). There were more female participants (60.3%) than male (39.7%). Most of the participants were either single (50.6%) or married (47.9%). Only 1.5% of the participants were divorced. Most had a Bachelor (42.4%) and Master (32.6%) degree. About 61.5% of the participants were students, and another 30.8% were employed at the time of data collection (Table 1).

When asked about their reasons for being abroad, about three-quarters (76.7%) indicated that they were there for higher education and 9.5% reported that they had traveled for job purposes. When probed further about their studentship status, 72.2% of the participants who traveled for study said that they were receiving a government scholarship, and 19.7% were self-funded. Concerning the location of residence, the majority lived in North America (United States and Canada; 44.6%), and 34.3% lived in the UK and Ireland. The rest were scattered across Europe (4.8%), the Middle East (2.0%), Africa (11.2%), and Australasia (3.2%). About half of the respondents lived with family (50.0%), 15.4% lived with other people, and 34.6% lived alone. Up to 24.3% of those who lived alone indicated that they had adopted this living style because of the COVID-19 pandemic.

### 3.2. Level of Anxiety and Depression

Concerning anxiety and depression among the participants and based on their scores calculated from the GAD-7 and PHQ-9, 34.4% and 29.6% met the diagnostic criteria for depressive and anxiety symptoms, respectively. The average score for the PHQ-9 was 8.3 ± 5.4 and for the GAD-7 was 7.4 ± 5.5. Severity of these symptoms varied but the majority had mild symptoms of depression (41.1%) and anxiety (37.3%). Most of the participants (47.7%) considered that the depressive symptoms caused difficulties in their daily activities at home and in the workplace. Similarly, a majority of the participants (48.3%) with symptoms of anxiety also developed difficulties in performing several activities at home and at work (Table 2).

### 3.3. General Aspects of Participants’ MH

To further examine the effects of the COVID-19 pandemic on general aspects of the participants’ MH (Table 3), 35.2% of the participants indicated that there was a large change in their life routines and 26% considered that alterations in routine were enormous. Apparently, the majority of the participants were not fully satisfied with their abilities to adapt to the lifestyle changes associated with the outbreak. A proportion of them (i.e., 6.9%, 23.4%, and 39.1%) indicated that they were dissatisfied, simply satisfied, and partly satisfied, respectively, with their skills to adapt to the diverse changes caused by COVID-19. A proportion of 48.2% of the participants considered the pandemic to have had moderate to severe impact on their MH. Conversely, 11.3% reported no impact on their MH. About 7.9% experienced a MH condition, and 88.8% of participants believed that the pandemic triggered their symptoms to varying degrees; however, the majority of the participants experienced slight (34.6%) to moderate provocation (28.8%). When asked about their relationships with the people they lived with, 44.0% and 29.9% indicated that their relationships were often good or always good, respectively. The participants’ experience with the lockdown was mostly positive (57.1%).

### 3.4. Access to MH Support and Services

About 63.6% of the respondents believed that they had no need for MH services whereas 8.3% thought that they needed access to MH services in order to support their MH (Table 4). A total of 11.7% and 11.9% of the participants agreed or strongly agreed with the notion that they would require support for their MH in the next three months and 12 months, respectively. Only 16.2% of the total participants were aware of available MH services.

### 3.5. Participants Perception of Their Coping Abilities

About 75% of the participants agreed or strongly agreed that living with others during the pandemic tended to help them cope better with the adverse circumstances concerning the outbreak (Table 5). Conversely, 22.5% agreed or strongly agreed that staying alone would help them cope well with the effects of the pandemic. When asked about their confidence to cope if quarantine continued for six more months, their responses varied from good (30.7%), very good (19.3%), to excellent (15.3%). When asked about their abilities to cope with another lockdown, only 12.2% and 10.4% considered their coping abilities to be very good and excellent, respectively.

### 3.6. Participants’ Perception of Government Response

Participants provided their opinions concerning the Saudi government’s response to their citizens’ MH needs (Table 6). About 43.8% of the study participants returned home during the lockdown, and the majority (82.8%) thought that the government’s reaction toward Saudi citizens living abroad was indeed positive. In addition, 37.9% and 29.7% of the participants agreed and strongly agreed that the collective response of the government and the embassies had a positive impact on their stability and MH. Up to 72.7% of the participants who travelled home during the lockdown experienced less mental pressure after returning home. The stress level of participants while abroad but before contacting the embassy was considered to be slight (24.1%), moderate (28.3%), and mostly severe (40.3%). The majority of the participants considered the government’s repatriation of its citizens back home during the pandemic to be excellent (63.8%), and 61% were very satisfied with the procedures and services provided by the Saudi MOH since returning home.

### 3.7. Factors Associated with Anxiety and Depression

Various sociodemographic factors were strongly associated with the participants’ depression and anxiety. Age, social status, education, reasons for traveling abroad, and location of residence were significantly associated with depression symptoms (*p* < 0.05) while gender, social status, education, and location of residence showed statistically significant associations with anxiety symptoms (Table 7). Concerning depression, participants younger than 35 years were significantly more likely to experience depressive symptoms (*p* ≤ 0.05). Furthermore, those who were single or divorced had high school education or no education, traveled for study, and lived in the UK and Ireland had statistically significant association with depressive symptoms (<0.05).

With regard to anxiety, females were found to be at a greater risk of developing anxiety. A total of 32.6% of females developed anxiety symptoms as opposed to 25.1% of males (*p* ≤ 0.05). Single or divorced individuals were also more likely to develop anxiety symptoms (*p* ≤ 0.05). In addition, those who had little or no education and those who lived in the UK/Ireland significantly experienced anxiety symptoms (<0.05).

### 3.8. Participants’ Other Variables

There were several other participants’ factors beyond sociodemographic characteristics that were tested and found to have significant influence on the production of depressive and anxiety symptoms among the responders (<0.05) (Table 8). The variables having significant associations with anxiety and depression were considerable changes to their routines, variable dissatisfaction with their abilities to adapt to changes, having awful relationships with the people sharing their accommodation, lack of confidence to cope with extended quarantine of another six months, unable to cope with another lockdown, and belief that the pandemic had a substantial impact on their MH.

Concerning the participants’ other important variables, this study teased apart an independent risk factor associated with depressive and anxiety symptoms. Participants who had a history of a diagnosis with MH condition, who had symptoms of their MH condition worsen moderately to dramatically, a strong need for MH support in the next 3 and 12 months, unaware of available MH services, a negative experience with the lockdown, traveled home during the lockdown, experienced considerable stress prior to contacting the Saudi embassy, and quarantined upon returning home had a significant association with depressive symptoms (<0.05). On the other hand, participants who believed that they would need MH support for the next 3 to 12 months, unaware of available MH services, had a negative experience with the lockdown, traveled home during the lockdown, and experienced moderate to severe stress before contacting the Saudi embassy significantly experienced anxiety symptoms (<0.05). In fact, some similar risk factors were involved in the correlation of both depressive and anxiety symptoms or may be mixed symptoms of depression-anxiety symptoms.

## 4. Discussion

This study examined the epidemiology and impact of the COVID-19 pandemic on the mental health of Saudi citizens living abroad during the pandemic. Approximately 62% of participants were students, and the majority (77%) remained abroad, primarily for educational purposes. Our study identified individuals who were female, younger, single, divorced, or living alone to be the most negatively affected by the COVID-19 pandemic; these individuals developed various acute psychiatric symptoms comparable with other studies [24]. Therefore, these specific groups are in greater need of tailored, comfortable, and simple communication and follow-up with Saudi Arabian embassies and cultural missions worldwide. Additionally, the Ministry of Health needs to provide a MH consultation hotline for Saudi students stranded abroad during the COVD-19 pandemic. The MOH has established health care centers for 14-day quarantine for students returning from foreign countries. Furthermore, to reduce the risk of developing common mental disorders during the pandemic, especially for those outside the country, a support network and telehealth services for MH should be available and free to all Saudi Arabian citizens.

Individuals and family members experiencing the COVID-19 pandemic and its associated stressors are more likely to develop several disturbances and maladjustments. In the context of severe stresses, the family adjustment and accommodation resource (FAAR) model has discussed increasing family demands, meanings related to the situation, adaptation, and the capabilities of individuals to overcome different stresses [25]. Similarly, the COVID-19 pandemic is a highly stressful, cataclysmic event affecting all aspects of human life and encompassing all communities, societies, races, and cultures worldwide. The survey presented in this study is an extremely relevant and timely exploration of the needs and perceptions of Saudi students in foreign countries concerning the available resources, access to health services, governmental assistance, living situation, family, and social or community support systems. Significant associations with anxiety and depression where such support systems were not available were detected, partly corroborating the results of other studies [25,26]. We found that international students faced diverse hardships during the COVID-19 pandemic including limited access to health services. Living abroad as a student is associated with financial obligations, housing, and security, all of which place tremendous pressure and demands on the students and their families. These pressures are even more apparent during an event such as the COVID-19 pandemic, as found in the present study.

According to our study, before contacting Saudi embassies in host countries, the majority of participants (92%) reported variable levels of stress. More than 80% appreciated the response of the Saudi government and embassy to meet the MH needs of students undergoing quarantine abroad and in Saudi Arabia. As a result, approximately 70% of participants reported stable mental health, and approximately 73% perceived reduced mental stress after returning home. Approximately 60% of respondents reported that the government repatriation of citizens was excellent, and MOH procedures and services during quarantine were highly satisfactory. Overall, the support of the Saudi government and embassies helped to mentally stabilize Saudi citizens including students, who consequently adapted well to the continuing situation due to a considerable reduction in stress levels. These findings align with a commentary report highlighting the Saudi government’s efforts to reduce pandemic-related psychological trauma simply by providing individuals and businesses with $133 million and offering free health services, initiatives that were well-received by the citizens [27]. According to the FAAR model, the perception that resources are sufficient, as reflected in the Saudi narrative, impacts how an individual adjusts to a situation [25]. Conversely, maladaptive patterns may emerge in cases where resources are scarce.

Concerning anxiety and depression among the study participants, approximately 34% and 30% met the diagnostic criteria for depressive and anxiety symptoms, respectively, partially consistent with a previous study [28]. These results were inconsistent with a recent study conducted in the early days of the pandemic in Saudi Arabia [29]. In this study, Alkhamees et al. (2020) reported that 28.3% and 24% of participants drawn from the general population expressed moderate to severe depression and anxiety levels, respectively [29]. The increased levels of depression and anxiety reported in our study may be due to the sample uniqueness of students living alone in foreign countries with no apparent support systems during the COVID-19 pandemic.

The coping mechanisms of individuals are extremely important for not experiencing MH impacts from the COVID-19 pandemic. However, the COVID-19 pandemic has created many different levels of stress and has severely compromised global psychological well-being. The pandemic has also changed the individuals’ perception of and ability to cope with different adversities during lockdown and quarantine. According to our study, fewer than 50% of participants perceived the pandemic to have substantially impacted their mental health compared with a study reporting that 78% of respondents developed poor mental health well-being during the COVID-19 pandemic [30]. Furthermore, the majority of our study participants (60%) reported a major change to their routines, and most (63%) were satisfied with their ability to adapt to the changes associated with COVID-19. Several studies have reported various reactions to the constant uncertainties related to infection, anxiety, irritation, isolation, social distance, and loneliness that impaired well-being, quality of life, resilience, and contributed to poor MH [1,8,31,32].

Most countries globally have sought to control the spread of COVID-19 through social distancing, lockdowns, quarantine, self-isolation, promoting public facemask use, limiting crowds, regularly testing people for NCV2, and treating symptomatic people. According to our survey, the majority of respondents strongly expressed that living with others with whom they shared a good or nonconflictual relationship and access to family and social support during lockdown enhanced their ability to cope and successfully adapt to the overwhelming stress and impact of COVID-19, findings compatible with other studies [33]. Continuation of stress for more than six months due to any event including lockdown tends to have an adverse impact on the MH of individuals during the pandemic [8]. Changes to awareness, knowledge, and perception of the pandemic can considerably affect the MH and psychological well-being of individuals including causing individuals to become more easily provoked and irritated and bringing about behavioral transformations of individuals [34,35].

There is no physical health without mental health, and each affects the other. During pandemic events like COVID-19, this relationship is more evident, and a variable number of people require MH services. According to our study, 8–12% of respondents believed they required MH services on both a short- and long-term basis. At the same time, sixty-four felt no need for such services. However, delaying MH care services due to any reason including unawareness of MH service availability has been found to lead to chronicity and poor outcome; Panchal et al. (2020) reported MH deterioration in individuals who skipped or delayed health care during the COVID-19 pandemic. Limited access to MH care and substance use treatment was partly attributable to a shortage of MH professionals [31]. Two major implications of these findings are that people in need of MH services during a pandemic crisis should not delay consultation with MH experts, and government agencies should restore the shortage of MH providers.

## 5. Limitations

A limitation of this study is that it did not consider the role of other relevant variables in the relationship between the COVID-19 pandemic and mental status of citizens living abroad (e.g., working in the health care sector, working closely with patients suffering from COVID-19, living in countries with a high or low incidence of COVID-19, etc.). In addition, the study did not measure the duration between requesting help and receiving a response from the embassy or cultural mission, and this may explain some of the anxiety and depression symptoms detected in our study. Additionally, further exploration is needed to investigate how many people actually accessed different MH interventions such as group support webinars and virtual or hotline psychological or drug interventions. This might shed more light on our findings regarding peoples’ perceptions regarding their need for MH services. Moreover, the results may only reflect MH status during the COVID-19 pandemic. Therefore, follow-up studies with a larger sample size, which will comprehensively determine the short- and long-term MH outcomes, are needed in the future. The strength of this pilot survey is that it focused on Saudi students’ MH experiences and perceptions of the COVID-19 pandemic while living in foreign countries with or without their families.

## 6. Conclusions

In summary, the COVID-19 pandemic represents an unprecedented threat to global mental health. Two-thirds of study participants who were in foreign countries during the COVID-19 pandemic reported anxiety or depressive symptoms. Living away from family and friends was significantly associated with increased loneliness and psychological distress. Younger, single, or divorced individuals living alone were more likely to experience depressive symptoms, and females were more likely to experience symptoms of anxiety. Participants who had a negative impression of the lockdown and substantial routine changes experienced increased psychiatric symptoms. As such, this study tentatively suggests that the COVID-19 pandemic might lead to severe MH conditions. Therefore, more attention should be given to the MH of citizens/students stranded in foreign countries, especially during unprecedented fatal events such as pandemics. In addition, psychological and drug interventions directed toward the vulnerable population are necessary and greatly needed. Further studies, which comprehensively explore the long-term mental health outcomes of the ongoing COVID-19 pandemic among Saudi citizens living in foreign countries, are highly recommended.

## 7. Recommendations

The study findings indicated that specific groups such as younger student, singles, divorced, and living alone are impacted the most by this experience. It is recommended that these specific groups to get more tailored communication and follow-ups from the Saudi Arabian cultural mission and have individualized communication with each one to assess for specific needs. In addition, a peer system of support can be initiated by the Ministry through the different university and regional Saudi student clubs who can provide further information to students and provide them with resources in their regions. Knowing that there are individuals nearby in the area might ease the social isolation.

In addition, mental health hotlines for Saudi students can be provided through the Ministry of Health. There are already groups for those who are placed into the 14 days quarantine, and perhaps those who are still abroad might benefit from similar interventions.

We recommend that equal attention be paid to the mental health of the population during pandemics, where online mental services should be available, accessible and free to those in need to minimize the short-term and long-term effects of these disorders. A mental health campaign should also be organized by the responsible authorities to raise the awareness level of the population about mental health. We also recommend that further similar studies must be carried out in the same context.

## Figures and Tables

**Table 1:** Sociodemographic characteristics of the participants (*n* = 662).

Variables	Categories	Frequency	%
Age	<25 years	143	21.6
	25–34 years	424	64.0
	35–44 years	81	12.2
	45–54 years	9	1.4
	≥55 years	5	0.8
Gender	Male	263	39.7
	Female	399	60.3
Social status	Single	335	50.6
	Married	317	47.9
	Divorced	10	1.5
Education	High school diploma	120	18.1
	Bachelor’s	281	42.4
	Master’s	216	32.6
	PhD or equivalent	39	5.9
	None	6	0.9
Employment status	Employed	204	30.8
	Self-employed	12	1.8
	Student	407	61.5
	Retired	5	0.8
	Unemployed	34	5.1
Reasons for being abroad	Tourism	27	4.1
	Study	508	76.7
	Work	63	9.5
	Resident	2	0.3
	Companion	62	9.4
Studentship status (*n* = 508)	Self-funded student	100	19.7
	Student on government scholarship	367	72.2
	Recently graduated	41	8.1
Location of residence (by region)	Europe	32	4.8
	Middle East	13	2.0
	Australasia	21	3.2
	Africa	74	11.2
	North America	295	44.6
	UK and Ireland	227	34.3
Living status	Living alone	229	34.6
	Living with family	331	50.0
	Living with other people	102	15.4
Live alone because of the pandemic	Yes	54	24.3
	No	168	75.7

**Table 2:** Prevalence and levels of depression and anxiety symptoms.

Variables	Depression	Anxiety
Score, Mean ± SD	8.34 ± 5.4	7.38 ± 5.5
Meet criteria for diagnosis, *n* (%)		
Yes	228 (34.4%)	196 (29.6%)
No	434 (65.6%)	466 (70.4%)
Severity, *n* (%)		
Minimal	162 (24.5%)	219 (33.1%)
Mild	272 (41.1%)	247 (37.3%)
Moderate	141 (21.3%)	114 (17.2%)
Moderately severe	56 (8.5%)	NA
Severe	31 (4.7%)	82 (12.4%)
Impact on activities at home and work		
Not difficult at all	214 (32.3%)	193 (29.2%)
Somehow difficult	316 (47.7%)	320 (48.3%)
Very difficult	132 (19.9%)	149 (22.5%)

**Table 3:** Effect of the pandemic on general aspects of the participants’ MH.

Variables	Values	Frequency	%
How much participant routines changed during the pandemic	No change	7	1.1
	Slight change	57	8.6
	Moderate change	193	29.2
	Big change	233	35.2
	Very big change	172	26.0
Satisfaction with ability to adapt to changes associated with the outbreak	Dissatisfied	46	6.9
	Simply satisfied	155	23.4
	Partly satisfied	259	39.1
	Satisfied	139	21.0
	Very satisfied	63	9.5
Relationship with people study participants live with (*n* = 541)	Bad all the time	5	0.9
	Bad most of the time	21	3.9
	Neither good nor bad	115	21.3
	Often good	238	44.0
	Always good	162	29.9
Effect of the pandemic on their mental health	No impact	75	11.3
	Slight impact	205	31.0
	Moderate impact	209	31.6
	Big impact	110	16.6
	Major impact	63	9.5
Participants experienced a mental health condition	Yes	52	7.9
	No	588	88.8
	N/A	22	3.3
Symptoms worsen during the pandemic (*n* = 52)	Symptoms never got worse	10	19.2
	Provoked slightly	18	34.6
	Provoked moderately	15	28.8
	Provoked dramatically	7	13.5
	Provoked very dramatically	2	3.8
Experience with the lockdown	Positive	378	57.1
	Negative	142	21.5
	I don’t know	142	21.5

**Table 4:** Access to mental health services and support during the pandemic.

Variables	Values	Frequency	%
Access to MH services needed to support MH	Yes	55	8.3
	No	186	28.1
	No need for MH services	421	63.6
Need support with MH in the next 3 months	Strongly disagree	132	19.9
	Disagree	162	24.5
	I don’t know	291	44.0
	Agree	64	9.7
	Strongly agree	13	2.0
Need support with MH in the next 12 months	Strongly disagree	130	19.6
	Disagree	108	16.3
	I don’t know	345	52.1
	Agree	63	9.5
	Strongly agree	16	2.4
Awareness of available MH services	Yes	107	16.2
	No	555	83.8

**Table 5:** Participants’ perception of their ability to deal with the MH impact of the pandemic.

Variables	Values	Frequency	%
Living with others during the pandemic has a good impact on ability to cope with the circumstances	Strongly disagree	9	1.4
	Disagree	30	4.5
	Neutral	125	18.9
	Agree	257	38.8
	Strongly agree	241	36.4
Being alone during the pandemic has a good impact on ability to cope with the effects of the pandemic	Strongly disagree	129	19.5
	Disagree	179	27.0
	Neutral	205	31.0
	Agree	104	15.7
	Strongly agree	45	6.8
Participants’ confidence in their ability to cope if quarantine continues for another 6 months	Weak	65	9.8
	Acceptable	165	24.9
	Good	203	30.7
	Very good	128	19.3
	Excellent	101	15.3
Ability to cope with another lockdown	Weak	173	26.1
	Acceptable	187	28.2
	Good	152	23.0
	Very good	81	12.2
	Excellent	69	10.4

**Table 6:** Participants’ perception of the Saudi government response to citizens abroad and their MH needs.

Variables	Values	Frequency	%
Traveled home during the lockdown	Yes	290	43.8
	No	372	56.2
Government reaction towards citizens in host countries (*n* = 290)	Positive	240	82.8
	Negative	50	17.2
Government and embassy response towards citizens outside had an impact on your stability and mental health (*n* = 290)	Strongly disagree	21	7.2
	Disagree	33	11.4
	I don’t know	40	13.8
	Agree	110	37.9
	Strongly agree	86	29.7
Experience less mental pressure since you returned home (*n* = 290)	Strongly disagree	10	3.4
	Disagree	40	13.8
	I don’t know	29	10.0
	Agree	112	38.6
	Strongly agree	99	34.1
Stress level while abroad before contacting the embassy (*n* = 290)	No stress	21	7.2
	Slight stress	70	24.1
	Moderate stress	82	28.3
	Severe stress	117	40.3
Participant evaluation of government repatriation arrangements for citizens (*n* = 290)	Weak	6	2.1
	Acceptable	15	5.2
	Neutral	7	2.4
	Good	34	11.7
	Very good	43	14.8
	Excellent	185	63.8
Participants quarantined (*n* = 290)	Yes	228	78.6
	No	62	21.4
Satisfaction with the procedures and services of the Saudi Ministry of Health (*n* = 228)	Not satisfied	7	3.1
	Neutral	18	7.9
	Satisfied	64	28.1
	Very satisfied	139	61.0

**Table 7:** Relationships between sociodemographic factors and each of depression and anxiety.

Variables	*n*	Depression			Anxiety		
		Yes	No	*p*	Yes	No	*p*
Age							
<35 years	567	206 (36.3%)	361 (63.7%)	**0.010**	171 (30.2%)	396 (69.8%)	0.45
35 and above	95	22 (23.2%)	73 (76.8%)		25 (26.3%)	70 (73.7%)	
Gender							
Male	263	79 (30.0%)	184 (70.0%)	0.050	66 (25.1%)	197 (74.9%)	**0.04**
Female	399	149 (37.3%)	250 (62.7%)		130 (32.6%)	269 (67.4%)	
Social status							
Single/divorced	345	141 (40.9%)	204 (59.1%)	**0.000**	120 (34.8%)	225 (65.2%)	**0.02**
Married	317	87 (27.4%)	230 (72.6%)		76 (24.0%)	241 (76.0%)	
Education							
High school diploma or no education	126	56 (44.4%)	70 (55.6%)	**0.009**	47 (37.3%)	79 (62.7%)	**0.04**
Bachelors to PhD	536	172 (32.1%)	364 (67.9%)		149 (27.8%)	387 (72.2%)	
Employment status							
Employed/student	623	216 (34.7%)	407 (65.3%)	0.620	182 (29.2%)	441 (70.8%)	0.38
Unemployed/retired	39	12 (30.8%)	27 (69.2%)		14 (35.9%)	25 (64.1%)	
Reasons for being abroad							
Work, tourism, others	154	38 (24.7%)	116 (75.3%)	**0.040**	42 (27.3%)	112 (72.7%)	0.47
Study	508	190 (37.4%)	318 (62.6%)		154 (30.3%)	354 (69.7%)	
Studentship status							
Self-funded student	100	33 (33.0%)	67 (67.0%)	0.260	33 (33.0%)	67 (67.0%)	0.77
Student on government scholarship	367	145 (39.5%)	222 (60.5%)		108 (29.4%)	259 (70.6%)	
Location of residence							
N. America	295	95 (32.2%)	200 (67.8%)	**0.010**	71 (24.1%)	224 (75.9%)	**0.02**
UK and Ireland	227	98 (43.2%)	129 (56.8%)		86 (37.9%)	141 (62.1%)	
Other places	140	35 (25.0%)	105 (75.0%)		39 (27.9%)	101 (72.1%)	
Living status							
Living alone	229	87 (38.0%)	142 (62.0%)	0.160	78 (34.1%)	151 65.9%)	0.07
Living with people (family/others)	433	141 (32.6%)	292 (67.4%)		118 (27.3%)	315 (72.7%)	
Living alone							
Yes	54	23 (42.6%)	31 (57.4%)	0.560	18 (33.3%)	36 (66.7%)	0.87
No	168	64 (38.1%)	104 (61.9%)		58 (34.5%)	110 (65.5%)	

Bolded *p*-values are significant at *p* < 0.05.

**Table 8:** Relationships between the participants’ other factors and each of depression and anxiety.

Variables	*n*	Depression			Anxiety		
		Yes	No	*p*	Yes	No	*p*
How much the participants’ routines changed during the pandemic							
No to moderate change	257	50 (19.5%)	207 (80.5%)	**0.000**	56 (21.8%)	201 (78.2%)	**0.000**
Big/very big change	405	178 (44.0%)	227 (56.0%)		140 (34.6%)	265 (65.4%)	
Satisfaction with ability to adapt to changes associated with the outbreak							
Dissatisfied to partly satisfied	460	194 (42.2%)	266 (57.8%)	**0.000**	161 (35.0%)	299 (65.0%)	**0.000**
Satisfied/very satisfied	202	34 (16.8%)	168 (83.2%)		35 (17.3%)	167 (82.7%)	
Relationship with people the study participants lived with							
Neutral to Bad all the time	141	65 (46.1%)	76 (53.9%)	**0.000**	60 (42.6%)	81 (57.4%)	**0.000**
Often/always good	400	113 (28.3%)	287 (71.8%)		94 (23.5%)	306 (76.5%)	
Participants’ confidence in their ability to cope if quarantine continued for another 6 months							
Weak or acceptable	230	108 (47.0%)	122 (53.0%)	**0.000**	97 (42.2%)	133 (57.8%)	**0.000**
Good to excellent	432	120 (27.8%)	312 (72.2%)		99 (22.9%)	333 (77.1%)	
Ability to cope with another lockdown							
Weak or acceptable	360	158 (43.9%)	202 (56.1%)	**0.000**	141 (39.2%)	219 (60.8%)	**0.000**
Good to excellent	302	70 (23.2%)	232 (76.8%)		55 (18.2%)	247 (81.8%)	
Effect of the pandemic on MH							
No to slight impact	280	41 (14.6%)	239 (85.4%)	**0.000**	41 (14.6%)	239 (85.4%)	**0.000**
Moderate to major impact	382	187 (49.0%)	195 (51.0%)		155 (40.6%)	227 (59.4%)	
Previous diagnosis of a mental health condition							
Yes	52	28 (53.8%)	24 (46.2%)	**0.001**	19 (36.5%)	33 (63.5%)	0.195
No	588	186 (31.6%)	402 (68.4%)		165 (28.1%)	423 (71.9%)	
Symptoms worsen during the pandemic							
Symptoms unprovoked or slightly provoked.	28	11 (39.3%)	17 (60.7%)	**0.023**	7 (25.0%)	21 (75.0%)	0.062
Moderate to very dramatic provocation	24	17 (70.8%)	7 (29.2%)		12 (50.0%)	12 (50.0%)	
Need support with mental health in the next 3 months							
Don’t know to strongly disagree	585	175 (29.9%)	410 (70.1%)	**0.000**	153 (26.2%)	432 (73.8%)	**0.000**
Agree/strongly agree	77	53 (68.8%)	24 (31.2%)		43 (55.8%)	34 (44.2%)	
Need support with mental health in the next 12 months							
Don’t know to strongly disagree	583	172 (29.5%)	411 (70.5%)	**0.000**	156 (26.8%)	427 (73.2%)	**0.000**
Agree/strongly agree	79	56 (70.9%)	23 (29.1%)		40 (50.6%)	39 (49.4%)	
Aware of available mental health services							
Yes	107	26 (24.3%)	81 (75.7%)	**0.016**	23 (21.5%)	84 (78.5%)	**0.045**
No	555	202 (36.4%)	353 (63.6%)		173 (31.2%)	382 (68.8%)	
Experience with the lockdown							
Positive	378	81 (21.4%)	297 (78.6%)	**0.000**	72 (19.0%)	306 (81.0%)	**0.000**
Negative/don’t know	284	147 (51.8%)	137 (48.2%)		124 (43.7%)	160 (56.3%)	
Traveled home during the lockdown							
Yes	290	113 (39.0%)	177 (61.0%)	**0.031**	104 (35.9%)	186 (64.1%)	**0.002**
No	372	115 (30.9%)	257 (69.1%)		92 (24.7%)	280 (75.3%)	
Experienced less mental pressure since you returned home							
Don’t know to strongly disagree	79	27 (34.2%)	52 (65.8%)	0.306	25 (31.6%)	54 (68.4%)	0.36
Agree/strongly agree	211	86 (40.8%)	125 (59.2%)		79 (37.4%)	132 (62.6%)	
Stress level while abroad before contacting the embassy							
No to slight stress	91	19 (20.9%)	72 (79.1%)	**0.000**	19 (20.9%)	72 (79.1%)	**0.000**
Moderate to severe stress	199	94 (47.2%)	105 (52.8%)		85 (42.7%)	114 (57.3%)	
Participants quarantined							
Yes	28	96 (42.1%)	132 (57.9%)	**0.036**	87 (38.2%)	141 (61.8%)	0.118
No	62	17 (27.4%)	45 (72.6%)		17 (27.4%)	45 (72.6%)	
Satisfaction with the procedures and services of the Saudi Ministry of Health							
Not satisfied or neutral	25	11 (44.0%)	14 (56.0%)	0.839	10 (40.0%)	15 (60.0%)	0.841
Satisfied/very satisfied	203	85 (41.9%)	118 (58.1%)		77 (37.9%)	126 (62.1%)	

Bolded *p*-values are significant at *p* < 0.05.

## Data Availability

The data that support the findings of this study are available from the corresponding author, [A.A.A.], upon reasonable request.
